# GCN-based unsupervised community detection with refined structure centers and expanded pseudo-labeled set

**DOI:** 10.1371/journal.pone.0327022

**Published:** 2025-07-01

**Authors:** Bing Guo, Liping Deng, Tao Lian

**Affiliations:** 1 Department of Computer Science and Technology, Taiyuan Normal University, Jinzhong, Shanxi, China; 2 College of Computer Engineering, Shanxi Vocational University of Engineering Science and Technology, Jinzhong, Shanxi, China; 3 College of Artificial Intelligence, Taiyuan University of Technology, Jinzhong, Shanxi, China; ICFAI Foundation for Higher Education Faculty of Science and Technology, INDIA

## Abstract

Community detection is a classical problem for analyzing the structures of various graph-structured data. An efficient approach is to expand the community structure from a few structure centers based on the graph topology. Considering them as pseudo-labeled nodes, graph convolutional network (GCN) is recently exploited to realize unsupervised community detection. However, the results are highly dependent on initial structure centers. Moreover, a shallow GCN cannot effectively propagate a limited amount of label information to the entire graph, since the graph convolution is a localized filter. In this paper, we develop a GCN-based unsupervised community detection method with structure center Refinement and pseudo-labeled set Expansion (RE-GCN), considering both the network topology and node attributes. To reduce the adverse effect of inappropriate structure centers, we iteratively refine them by alternating between two steps: obtaining a temporary graph partition by a GCN trained with the current structure centers; updating each structure center to the node with the highest structure importance in the corresponding induced subgraph. To improve the label propagation ability of shallow GCN, we expand the pseudo-labeled set by selecting a few nodes whose affiliation strengths to a community are similar to that of its structure center. The final GCN is trained with the expanded pseudo-labeled set to realize community detection. Extensive experiments demonstrate the effectiveness of the proposed approach on both attributed and non-attributed networks. The refinement process yields a set of more representative structure centers, and the community detection performance of GCN improves as the number of pseudo-labeled nodes increase.

## Introduction

Many complex systems in the real world can be abstracted as a network [1], e.g., social networks, biological networks, and citation networks. Sometimes, the nodes in these networks are also attached with abundant attributes. A prominent feature of various networks is the existence of community structure [2–4]—the organization of nodes into groups, where nodes in the same group are densely connected or share similar attributes. Community detection is helpful to reveal mesoscale properties of complex networks [5,6]. For example, it can facilitate the detection of protein complexes and functional modules in protein-protein interaction networks [7].

Many existing research explores the community structure from the global view [8], which takes the entire network as a whole and optimizes some global quality function. Typical global methods include modularity maximization [9–11], spectral clustering [12], hierarchical clustering [2,13], etc. Global methods are often computationally expensive and faced with the resolution limit [14] that prevents them from identifying small communities in large networks. Moreover, sometimes one might only care about communities in a small region, which should have little to do with portions of the network that are very far away [8].

An alternative approach is local community detection, which only utilizes local information to build individual communities around a few seed nodes [15–19]. These methods are computationally efficient without the need to analyze the entire network. Among them, local expansion methods are widely used for local community detection in large networks. Such methods build a local community around a specified seed node by adding nodes greedily into the community until a local optimum of some quality function is reached [20,21]. However, a few seed nodes are required to be specified in advance, and the results are sensitive to initial seeds [15]. To overcome this problem, Wang *et al*. [16] were inspired by the literature [22], and proposed a method to automatically identify the center of the network structure. These nodes are characterized by a higher local density than their neighboring nodes and a relatively large distance from other nodes with higher density. They can be used as seed nodes for the local expansion method.

Recently, community detection through deep learning has received considerable attention [23]. In particular, graph convolutional network (GCN) is exploited in many works to realize community detection [17,18,24]. The graph convolution layer can be seen as a local filter that can efficiently propagate and aggregate the information of local neighbors to derive low-dimensional node representations, which are further used to infer their community labels. To train the GCN, only a few seed nodes [17] (or structure centers [18]) can be used as labeled (or pseudo-labeled) nodes. Hence, Wang *et al*. exploited the label propagation algorithm [25] to acquire a little extra supervision information [17], or simply added several neighbors of the structure centers into the training set [18].

However, the community detection performance of the above methods is hindered by two obstacles: inappropriate structure centers and insufficient propagation ability.

**Fig 1:**
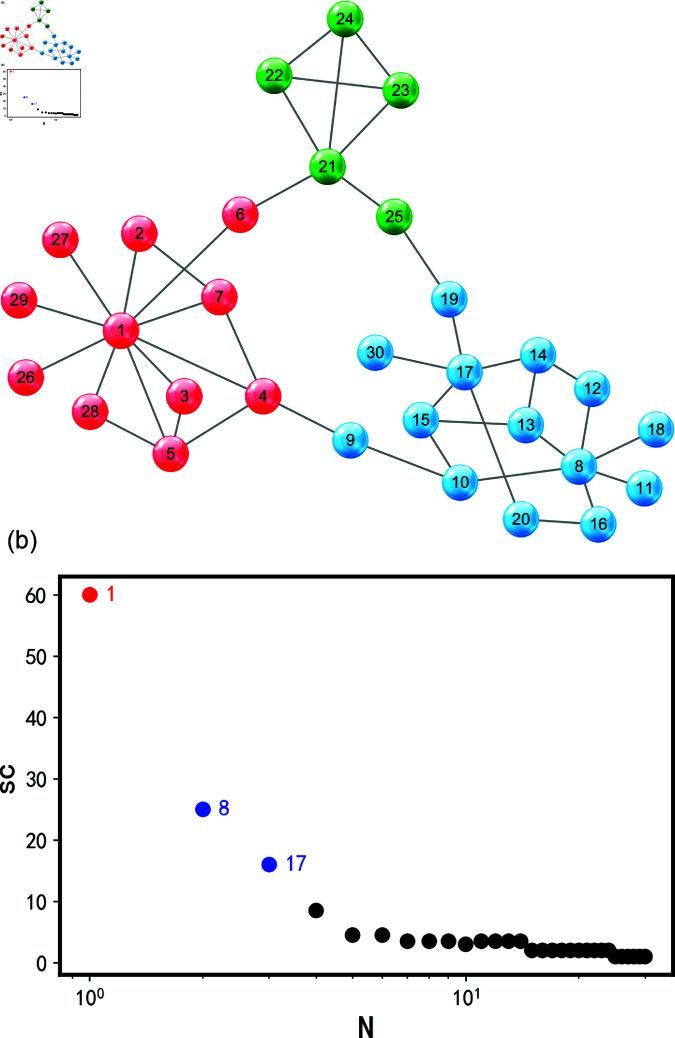
The identified structure centers may be problematic. (a) The ground-truth community partition (b) The structural centralities of different nodes.

The initial structure centers may be inappropriate, which has an adverse effect on the resulting communities. As shown in Fig 1, two of the three nodes with the largest structure centrality [22] belong to the same community, leaving out the nodes in the third ground-truth community. The reason is that the structure center selection procedure only exploits the network topology, ignoring the node attributes. Thus, it is necessary to refine the initial structure centers that serve as seed nodes for community detection.

GCN cannot effectively propagate the labels to the entire graph when given limited amount of supervision information [26]. As known, the graph convolution is a local filter that induces a node’s representation by aggregating its neighbors’ information. To avoid over-smoothing, shallow GCNs are widely used in the literature, which however has insufficient propagation ability on large networks with only a few seeds [26]. A larger and balanced set of pseudo-labeled nodes need to be constructed and fed into GCN as the supervision information.

**Fig 2:**
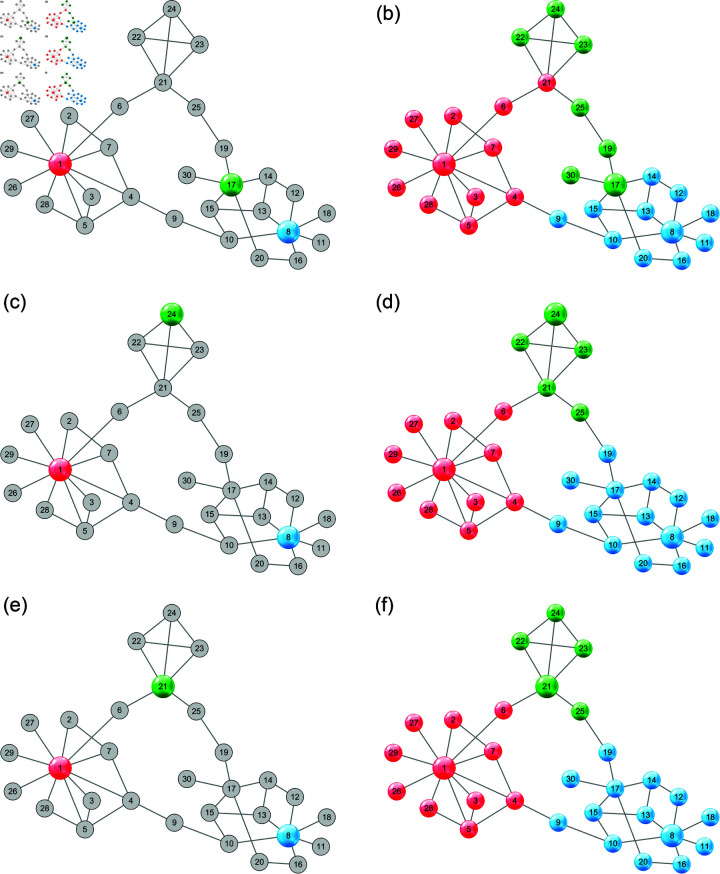
An example network showing the iterative refinement of structure centers and graph partition. (a) Initial structure centers (b) Graph partition in 1st pass (c) Updated structure centers (d) Graph partition in 2nd pass (e) Updated structure centers (f) Graph partition in 3rd pass.

To overcome these two problems, we propose a novel unsupervised approach to community detection based on GCN, which leverages both graph topology and node attributes to refine the structure centers and expand the set of pseudo-labeled nodes. It firstly identifies a few structure centers that have high local density and are far away from each other. To reduce the adverse effect of inappropriate structure centers, we iteratively refine the initial structure centers by alternating between two steps: obtaining a temporary graph partition by training a GCN with the current structure centers; updating each structure center to the node with the highest structure importance in the corresponding induced subgraph. The process is shown in Fig 2 with an example network. The initial structure centers (i.e., nodes 1, 8, and 17) in Fig 2(a) are updated to a set of more representative seeds (i.e., nodes 1, 8, and 21) in Fig 2(e). For larger networks, a GCN trained only with these few structure centers is not able to make accurate predictions for all the remaining nodes. To make up for the lack of propagation ability, we construct a larger and balanced pseudo-labeled set by selecting several nodes whose affiliation strength to a community is similar to that of its structure center. The final GCN is trained with the expanded pseudo-labeled set to realize community detection.

The main contributions of this paper are summarized as follows.

- We propose an unsupervised approach to community detection based on GCN which can leverage both network topology and node attributes, and demonstrate its effectiveness on both attributed and non-attributed networks.
- We develop an iterative structure center refinement strategy which can yield a better set of proper structure centers and lay a good foundation for community detection.
- We devise a pseudo-labeled set expansion strategy based on community affiliation strength which can make up for the lack of propagation ability of shallow GCN by supplying it with a larger amount of supervision information.

The rest of this paper is organized as follows. In section Related Work, we introduce related work on community detection and graph convolutional network. In section Preliminaries, we present the problem formulation along with other preliminaries. In section Methodology, we elaborate the proposed approach in detail. Experiment results and analysis are presented in section Experiments, followed by concluding remarks in section Conclusion.

## Related work

In this section, we present the related work on community detection and graph convolutional networks.

### Community detection

Community detection is one of the important tasks in network data mining that helps us to analyze and understand the structural properties and group characteristics of various networks. Existing research attempts to explore the community structure either from the global view or from the local view.

Global methods require information about the whole network structure and partitioning it from the global perspective [8]. Recently, some scholars have designed nice clustering algorithm frameworks, where the type of graph, and the sparsity and noise of the initial graph are fully considered before clustering, in addition to multi-scale information embedding learning (multi-scale information embedded), all these factors affect the clustering effect. For example, the scholars [27] propose methods that can cluster homomorphic and heteromorphic graphs simultaneously. First, homomorphic and heteromorphic graphs are constructed separately, then the two graphs are fused into a single graph, and finally the attributes and structures of this graph are encoded and learned. Based on this, the scholars [28] propose a novel method, namely deep attention-guided graph clustering with dual selfsupervision (DAGC). Inspired by the success of Variational Graph Auto-Encoders (VGAEs) learning, the article [29] addresses improvements to Variational Graph Auto-Encoders (VGAEs) type methods: they formulate a new variational lower bound that incorporates an explicit clustering objective function. To improve the clustering performance, the article [30] proposes a novel clustering network called Embedding-Induced Graph Refinement Clustering Network (EGRC-Net), which effectively utilizes the learned embedding to adaptively refine the initial graph and enhance the clustering performance. Typical methods include modularity maximization [9–11], spectral clustering [12], hierarchical clustering [2,13], etc. The global approach has several limitations. For example, it is time-consuming to compute the eigen vectors in spectral clustering for large networks. Modularity optimization may fail to identify communities smaller than a scale, i.e., the resolution limit [14]. Moreover, it is hard to know the entire network in real settings, which is also unnecessary if the user only wants to know the local community structure in a small region of a huge network.

Compared with global methods, local methods can effectively discover communities without complete information about the entire network [27]. A widely used approach is to start from a few seed nodes and expand them into several local communities [15–17]. Such methods can be parallelized and are scalable to large networks. However, local expansion methods only perform well when the seed nodes are located in the core region of individual communities, which is known as the seed-dependence problem. To alleviate this issue, several studies make efforts to select a good set of seeds [15,16,30,31]. For instance, Chen *et al*. [30] considered the node with local maximum degree as a better starting node. Inspired by Rodriguez and Laio [22], Wang *et al*. [16] recently proposed the structural centrality index to identify structural centers in a network that have a higher local density than their neighbors and a relatively large distance from other nodes with higher densities. But inappropriate nodes may be identified as structure centers, since the above method only exploits the network topology, ignoring the node attributes.

### GCN-based community detection

In recent years, different types of graph neural networks [29,32,33] have been proposed to boost the performance of various graph analysis tasks. In particular, Graph Convolutional Network (GCN) [32] is a successful attempt of generalizing the powerful convolution operation from Euclidean data to graph-structured data, which can effectively integrate the network topology and node attributes to extract deeper network features. However, traditional GCNs often assume isotropic information propagation, neglecting directional relationships that could better capture community structures [34]. Variants of GCN have shown excellent performance in different tasks, such as node classification [35], personalized recommendation [36], and traffic prediction [37]. It’s worth noting that traditional graph clustering methods like symmetric nonnegative matrix factorization [38] still provide valuable theoretical foundations for modern GNN-based approaches.

Without exception, GCN is also applied to the problem of community detection on complex networks. For example, Jin *et al*. [24] integrated GCN and MRF to realize semi-supervised community detection. Note that semi-supervised learning for GCN requires a considerable amount of labeled nodes to achieve satisfying performance [26], while recent semi-supervised deep attributed clustering methods [39] have shown promising results in reducing annotation dependency. However, it is hard to acquire enough high-quality labeled nodes for community detection in large networks.. However, it is hard to acquire enough high-quality labeled nodes for community detection in large networks. Hence, several GCN based unsupervised methods have been proposed for community detection [40–42]. While spectral clustering with graph learning [43] represents another important direction, our work focuses specifically on GCN-based architectures. Among them, graph autoencoder is a commonly adopted architecture [29,40,41], where a graph convolutional module is used as the encoder to obtain latent node representations, which are then passed through the decoder to minimize the reconstruction error for the graph adjacency matrix (as well as node attributes). The learned node representations are finally used to infer their community labels by node clustering. The CLEAR model [44] is a novel unsupervised GNN model with cluster-aware self-training, which learns embeddings using intrinsic network cluster properties and thus needs no direct supervision from labels. Second, unlike other GNN models that rely on a static graph structure, CLEAR further proposes a topology refining scheme that reduces inter-cluster connections of neighbor nodes to alleviate the impact of noisy edges. However, the model refinement process only considers the topology ignoring the node attributes, and does not account for anomalous edge patterns that may distort community detection [45].

## Preliminaries

This section first presents our problem formulation along with basic notations, and then introduces the essentials of graph convolutional network used in our model.

### Problem formulation

We are interested in the community detection task in an undirected graph 𝒢=(𝒱,ℰ), where $𝒱=\{v_{1},v_{2},\cdots ,v_{N}\}$ denotes the set of nodes, and ℰ denotes the set of edges. The topology of 𝒢 can be represented by its adjacency matrix $𝐀\in {\mathbb{R}}^{N\times N}$. In settings where the nodes in 𝒢 are associated with attributes, $𝐗\in {\mathbb{R}}^{N\times F}$ is used to denote the feature matrix, where the *i*-th row, i.e., ${𝐱}_{i}={𝐗}_{i*}$, is the feature vector for node $v_{i}$. In this paper, we focus on the problem of non-overlapping community detection based on the network topology **A** and node attributes **X**, which aims to partition the node set 𝒱 into a set of disjoint communities $𝒞=\{{𝒞}_{1},{𝒞}_{2},\ldots ,{𝒞}_{K}\}$, where ${𝒞}_{i}\subseteq 𝒱$ for i=1,2,…,K and $\overset{K}{\underset{i=1}{⋃}}{𝒞}_{i}=𝒱$. We treat it as an unsupervised learning task, where the ground-truth community label for each node is not available for training.

### Graph convolutional network

To make full use of network topology and attribute information, GCN is a basic module in our method. GCN [32] is a multi-layer neural network that operates directly on a homogeneous graph and induces the embedding vector of a node based on the properties of its neighbors. The layer-wise propagation rule is as follows:

$${𝐇}^{\left(l+1\right)}=\sigma \left({\tilde{𝐃}}^{-\frac{1}{2}}\tilde{𝐀}{\tilde{𝐃}}^{-\frac{1}{2}}{𝐇}^{\left(l\right)}{𝐖}^{\left(l\right)}\right).$$ (1)

It is a special form of localized filter—a linear combination of the feature vectors of adjacent neighbors. $\tilde{𝐀}=𝐀+𝐈$, which added self-connections to the original adjacency matrix. $\tilde{𝐃}$ be the degree matrix, where ${\tilde{𝐃}}_{ii}=\sum_{j}{\tilde{𝐀}}_{ij}$. ${𝐖}^{\left(l\right)}$ is a layer-specific trainable transformation matrix. σ(.) denotes an activation function such as ReLU. ${𝐇}^{\left(l\right)}$ denotes the hidden representations of nodes in the *l*-th layer. Initially, ${𝐇}^{\left(0\right)}=𝐗$. For non-attributed networks, **X** is initialized as one-hot representations of nodes in the graph.

## Methodology

### Overview

**Fig 3:**
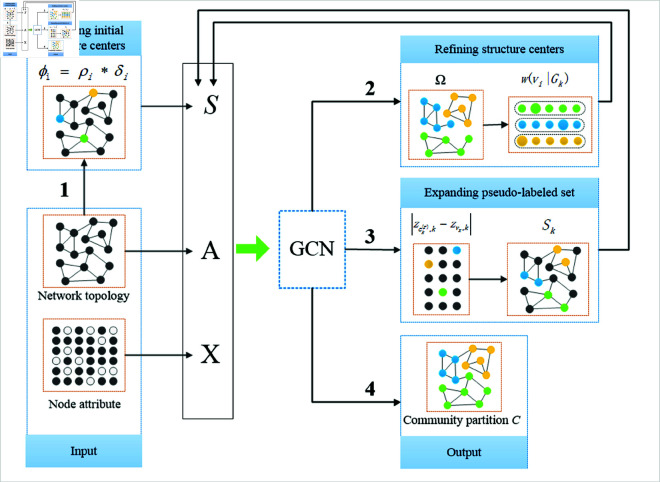
RE-GCN is comprised of four steps: 1) selecting initial structure centers; 2) refining the structure centers; 3) expanding pseudo-labeled set; and 4) finally training GCN for community partition.

The proposed model framework is shown in Fig 3. Firstly, we select a few structure centers based on graph topology. Secondly, initial structure centers are iteratively updated by considering both the network topology and node attributes. The refined structure centers can be regarded as representatives of different communities, and constitute a small set of pseudo-labeled nodes—one per community. Thirdly, we assign more nodes with pseudo community labels based on temporary partition, yielding a larger training set of pseudo-labeled nodes. With the expanded pseudo-labeled training set, the GCN can be trained to predict the community labels for the remaining nodes.

### Selecting initial structure centers

The selection of initial structure centers is particularly important. As the carrier of initial labels, they affect the resulting communities to a certain extent. A structure center should have high local density and meanwhile keep a relatively large distance from other nodes with higher density. Thus, the structural centrality of a node should take into account two aspects: the local density and the relative distance [22]. In the following, we present the corresponding definitions.

*Definition 1: Local Density.*

The local density of node $v_{i}$ in the network is defined as:

$$\rho_{i}=\sum_{j}ℍ\left(d_{c}-d_{ij}\right),$$ (2)

where *d*_*ij*_ denotes the distance between node $v_{i}$ and $v_{j}$, and *d*_*c*_ is a cutoff distance. ℍ(t) is the Heaviside step function.

$$ℍ\left(t\right)=\left\{ \begin{array}{cc} 1, & t>=0;\\ 0, & t<0. \end{array}\right.$$ (3)

Intuitively, $\rho_{i}$ is equal to the number of nodes with a distance shorter than *d*_*c*_ to node $v_{i}$. When *d*_*c*_ = 1, $\rho_{i}$ is equal to the number of nodes directly connected to node $v_{i}$, i.e., its degree.

*Definition 2: Relative Distance.*

The relative distance is measured by computing the minimum distance between node $v_{i}$ and any other nodes with higher local density.

$$\delta_{i}=\min_{j:\rho_{j}>\rho_{i}}d_{ij}.$$ (4)

If node $v_{i}$ has the highest local density, since there is no node $v_{j}$ with larger density $\rho_{j}$ than $\rho_{i}$, $\delta_{i}=\max_{j}d_{ij}$. Note that $\delta_{i}$ is much larger than typical nearest neighbor distances only for nodes with a local maximum density.

*Definition 3: Structural Centrality.*

A structural center should not only have a higher density than its neighbors, but also have a relatively large distance from other nodes with higher local density. The structural centrality of node $v_{i}$ is defined as:

$$\phi_{i}=\rho_{i}\cdot \delta_{i}.$$ (5)

The requirement of a relativly large $\delta_{i}$ could avoid the situation that multiple nodes with high local density in the same community are simultaneously identified as the structure centers to some extent.

The procedure for selecting initial structure centers is listed in algorithm 1, which selects the *K* nodes with largest centrality and assigns them with distinct community labels 1,2,…,K respectively. The output is denoted as ${𝒮}^{\left(0\right)}=\left\{\left\{c_{1}^{\left(0\right)}\right\},\left\{c_{2}^{\left(0\right)}\right\},\ldots ,\left\{c_{K}^{\left(0\right)}\right\}\right\}$, where $c_{k}^{\left(0\right)}$ denotes the *k*-th structure center and is assigned a pseudo community label *k*.

**Algorithm 1 Initial structure centers selection.**



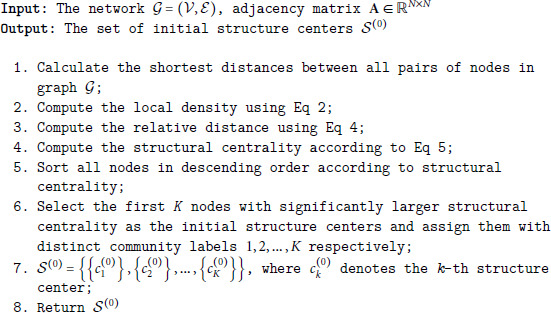



### Refining structure centers

Now we have identified *K* structure centers by algorithm 1. Ideally, for each ground-truth community, there is one structure center belonging to it, which could be regarded as a representative node for the community. In practice, sometimes none of the nodes in a community is identified as a structure center, and more than one structure centers might belong to the same ground-truth community, as shown in Fig 2(a). Hence, the results may be unsatisfactory: a small community may disappear if no node in it is identified as one of the initial structure centers; a large community might be split into more than two fragments if more than two nodes are identified as initial structure centers. The reason may be that the procedure for selecting initial structure centers only depends on the graph topology, ignoring the node attributes. Nevertheless, many real networks often exhibit the homophily principle [46]: nodes with similar attributes are more likely to connect to each other, forming a cohesive community.

To reduce the adverse effect of inappropriate structure centers, we propose to refine the initial structure centers by leveraging both graph topology and node attributes. Technically, we propose to refine the initial structure centers iteratively, as shown in algorithm 2. Firstly, we train a GCN to predict the community labels for each node, yielding a temporary partition of all nodes in the graph. Secondly, we build *K* induced subgraphs and refine the *k*-th structure center according to the structure of the *k*-th subgraph. The two steps are repeated until a stable state is reached—the structure centers stay unchanged between two consecutive iterations. Fig 2 visualizes the update process of structure centers for an example network.

Specifically, a two layer GCN is trained under the supervision of current structure centers ${𝒮}^{\left(0\right)}$. The GCN takes as input the graph adjacency matrix **A** and node attribute matrix **X**. The output, denoted as $𝐙\in {\mathbb{R}}^{N\times K}$, is computed as

$$𝐙=f\left(𝐗,𝐀\right)=\text{softmax}\left(\hat{𝐀}\;\text{ReLU}\left(\hat{𝐀}𝐗{𝐖}^{\left(0\right)}\right){𝐖}^{\left(1\right)}\right).$$ (6)

$\hat{𝐀}={\tilde{𝐃}}^{-\frac{1}{2}}\tilde{𝐀}{\tilde{𝐃}}^{-\frac{1}{2}}$, which is the normalized adjacency matrix with self connection. ${𝐖}^{\left(0\right)}\in {\mathbb{R}}^{F\times D}$ and ${𝐖}^{\left(1\right)}\in {\mathbb{R}}^{D\times K}$ are the weight parameters for the two graph convolution layers. ReLU(x)=max(0,x), and $\text{softmax}\left(x_{i}\right)=\frac{\exp\left(x_{i}\right)}{\sum_{i^{\prime }}\exp\left(x_{i^{\prime }}\right)}$, which are the activation functions for the first and second GCN layer. Let the entry *z*_*i*,*k*_ denote the affiliation strength of node $v_{i}$ to the *k*-th community. Then the predicted community label for an unlabeled node $v_{i}$ is

$${\hat{y}}_{i}=\underset{k\in \left[1,2,\ldots ,K\right]}{\arg\max}z_{i,k}.$$ (7)

Based on the predicted community labels, we can obtain a temporary partition of the nodes $\Omega =\left\{{𝒱}_{1},{𝒱}_{2},\ldots ,{𝒱}_{K}\right\}$, where ${𝒱}_{k}=\left\{v_{i}|{\hat{y}}_{i}=k\right\}$.

Then *K* subgraphs can be induced: ${𝒢}_{k}=\left({𝒱}_{k},{ℰ}_{k}\right)$, where ${ℰ}_{k}=\{\left(v_{i},v_{j}\right)|v_{i}\in {𝒱}_{k},v_{j}\in {𝒱}_{k},\left(v_{i},v_{j}\right)$ ∈ℰ}, for k=1,2,…,K. Some bad structure centers may emerge now: the *k*-th initial structure center, i.e., $c_{k}^{\left(0\right)}$, may locate on the periphery of subgraph ${𝒢}_{k}$ or even in another subgraph ${𝒢}_{k^{\prime }}$. To find the core of ${𝒢}_{k}$, we compute the local structure importance for each node in ${𝒢}_{k}$, which is calculated from the perspective of shortest path [47]. Formally speaking, we introduce the following definitions.

*Definition 4: SLP (Similarity based on Local Simple Paths).*

Given a network ${𝒢}_{k}=\left({𝒱}_{k},{ℰ}_{k}\right)$, the SLP between nodes $v_{i}$ and $v_{j}$ is defined as:

$$\text{SLP}\left(v_{i},v_{j}|{𝒢}_{k}\right)=\left\{ \begin{array}{cc} \alpha_{1}\eta_{i,j}^{\left(1\right)}+\alpha_{2}\eta_{i,j}^{\left(2\right)}+\alpha_{3}\eta_{i,j}^{\left(3\right)} & v_{i}\neq v_{j},\\ 0 & v_{i}=v_{j}. \end{array}\right.$$ (8)

$\eta_{i,j}^{\left(l\right)}$ is the number of simple paths—paths with no repeated nodes—with length *l* between nodes $v_{i}$ and $v_{j}$. $\alpha_{1},\alpha_{2},\alpha_{3}$ are non-negative weights that satisfy $\alpha_{1}\geq \alpha_{2}\geq \alpha_{3}$ and $\alpha_{1}+\alpha_{2}+\alpha_{3}=1$. Intuitively, a higher value of $\text{SLP}\left(v_{i},v_{j}|{𝒢}_{k}\right)$ indicates that the two nodes $v_{i}$ and $v_{j}$ are better connected in the subgraph ${𝒢}_{k}$.

*Definition 5: Local Structural Importance.*

Given a network ${𝒢}_{k}=\left({𝒱}_{k},{ℰ}_{k}\right)$, the notion of local structural importance of node $v_{i}\in {𝒱}_{k}$ is defined as:

$$w\left(v_{i}|{𝒢}_{k}\right)=\sum_{v_{j}\in {𝒱}_{k}}\text{SLP}\left(v_{i},v_{j}|{𝒢}_{k}\right).$$ (9)

Since $\text{SLP}\left(v_{i},v_{j}|{𝒢}_{k}\right)$ measures the connectivity strength between nodes $v_{i}$ and $v_{j}$ from the perspective of the number of simple paths shorter than 3, $w\left(v_{i}|{𝒢}_{k}\right)$ can indicate the local structure importance of node $v_{i}$ with respect to its surrounding nodes. A higher value of $w\left(v_{i}|{𝒢}_{k}\right)$ means that the node $v_{i}$ is closely connected to other nodes in the local neighborhood and is more likely to become a center of ${𝒢}_{k}$. Therefore, the node with the largest value of $w\left(v_{i}|{𝒢}_{k}\right)$ is selected as the structure center for the subgraph $w^{{𝒢}_{k}}$. That is to say,

$$c_{k}^{\left(r\right)}=\underset{v_{i}\in {𝒱}_{k}}{\arg\max}w\left(v_{i}|{𝒢}_{k}\right).$$ (10)

**Algorithm 2 Structure centers refinement.**



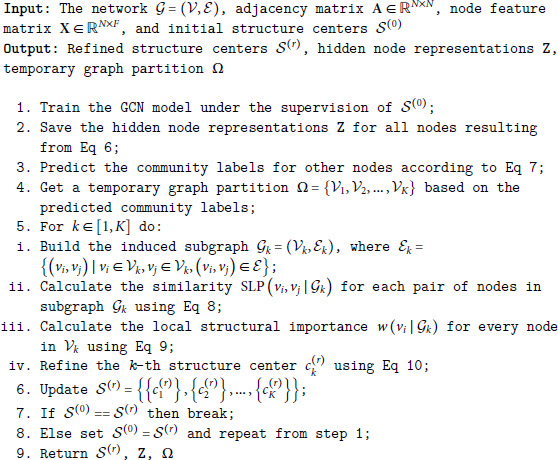



### Expanding pseudo-labeled set

Now we have obtained a set of *K* refined structure centers, each of which is assigned a distinct pseudo community label. To realize community partition, a GCN can be trained to predict the community labels for other nodes in the graph by integrating graph structure and node attributes. As known, a GCN is a localized filter, hence it cannot effectively propagate the label information to the entire graph when only a limited amount of labeled nodes are available [26]. In order to train a better GCN for community detection, we propose to expand the pseudo-labeled set by the following process.

When the structure centers are iteratively updated in Algorithm 2, a temporary graph partition $\Omega =\left\{{𝒱}_{1},{𝒱}_{2},\ldots ,{𝒱}_{K}\right\}$ is also returned: node $v_{i}$ is assigned to ${𝒱}_{k}$ if $k={\arg\max}_{k^{\prime }}z_{i,k^{\prime }}$. For each node $v_{x}\in {𝒱}_{k}$, we compute the difference between its affiliation strength to the *k*-th community and that of $c_{k}^{\left(r\right)}$, i.e., $\left|z_{v_{x},k}-z_{c_{k}^{\left(r\right)},k}\right|$. Then we construct the set of nodes ${𝒮}_{k}$ with pseudo label *k* by selecting the τ nodes with the smallest difference among ${𝒱}_{k}$.

$${𝒮}_{k}=\underset{v_{x}\in {𝒱}_{k}}{\arg\min}\left(\left|z_{v_{x},k}-z_{c_{k}^{\left(r\right)},k}\right|,\tau \right),$$ (11)

where argmin(·,τ) is the function to select the τ nodes with smallest values. The lower bound of τ is estimated by solving *K* · τ · ${\bar{d}}^{L}\approx N$, where $\bar{d}$ is the average degree of nodes in 𝒢, and *L* is the number of graph convolution layers which is 2 in our experiments. Note that the pseudo-label expansion process takes into account both the graph topology and node attributes, since the probability $z_{v_{x},k}$ for node $v_{x}$ given by a *L*-layer GCN depends on the labels and attributes of its *L*-hop neighbors in the graph, as defined in Eq 6. In this way, we expand the set of refined structure centers ${𝒮}^{\left(r\right)}=\left\{\left\{c_{1}^{\left(r\right)}\right\},\left\{c_{2}^{\left(r\right)}\right\},\ldots ,\left\{c_{K}^{\left(r\right)}\right\}\right\}$ to a larger set of pseudo-labeled nodes $𝒮=\left\{{𝒮}_{1},{𝒮}_{2},\ldots ,{𝒮}_{K}\right\}$.

An alternative strategy is to select the τ nodes with the largest *z*_*i*,*k*_ among all nodes in 𝒱 [26], i.e.,

$${𝒮}_{k}=\underset{v_{x}\in 𝒱}{\arg\max}\left(z_{v_{x},k},\tau \right).$$ (12)

We did not adopt this strategy, because it is sensitive to inappropriate structure centers. Besides, some nodes satisfying this criterion are ones of low degree which are located on the periphery of a certain community and far away from other communities. Although such nodes belong to the corresponding community with a high confidence, they have limited propagation ability.

### Training GCN with expanded pseudo-labeled set

Under the supervision of the larger set of pseudo-labeled nodes 𝒮, we can train a two-layer GCN with the same structure as defined by Eq 6. We adopt the cross-entropy loss over all pseudo-labeled nodes:

$$𝒥:=-\sum_{k=1}^{K}\sum_{v_{i}\in {𝒮}_{k}}\log z_{ik}$$ (13)

where ${𝒮}_{k}$ denotes the set of nodes with pseudo label *k*, and *K* is the output dimension of the softmax layer, which actually corresponds to the number of communities. The adam [48] optimizer is used to update the model parameters ${𝐖}^{\left(0\right)}$ and ${𝐖}^{\left(1\right)}$. Once the GCN is trained to convergence, we can predict the community label ${\hat{y}}_{i}$ for every node $v_{i}\in 𝒱$ using Eq 7 and obtain the final community partition $𝒞=\{{𝒞}_{1},{𝒞}_{2},\ldots ,{𝒞}_{K}\}$, where

$${𝒞}_{k}=\left\{v_{i}|{\hat{y}}_{i}=k\right\}\text{for }k=1,2,\ldots ,K.$$ (14)

The whole process of our proposed method is shown in in Algorithm 3.

**Algorithm 3 RE-GCN community detection.**



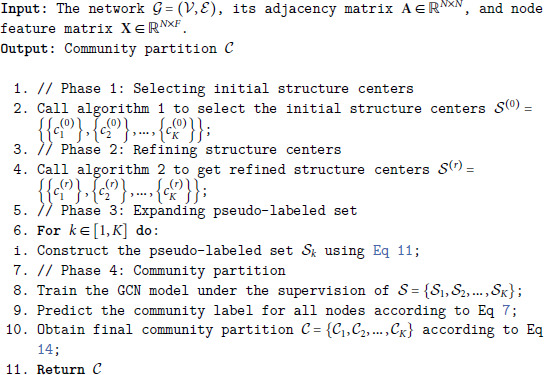



## Experiments

In this section, we validate the performance of the proposed community detection method on various real-world networks. We conduct extensive experiments with the aim of answering the following research questions:

RQ1: How well does the proposed RE-GCN perform in detecting communities on both attributed and non-attributed networks compared with other methods?
RQ2: Are both structure center refinement and pseudo-labeled set expansion essential for RE-GCN?
RQ3: Does the step of refining structure centers indeed yield better structure centers for later community detection?
RQ4: How do the expansion strategy and the size of pseudo-labeled set influence the community detection performance?

### Datasets

We conduct extensive experiments on 8 public network datasets, including Karate (http://konect.cc/networks/ucidata-zachary/), Dolphins (http://www-personal.umich.edu/~mejn/netdata/), Football (https://www.cc.gatech.edu/dimacs10/archive/clustering.shtml) PolBooks (https://www.cc.gatech.edu/dimacs10/archive/clustering.shtml), PolBlogs (https://www.cc.gatech.edu/dimacs10/archive/clustering.shtml), Cora (https://linqs.org/datasets/#cora), CiteSeer (https://linqs.org/datasets/#citeseer-doc-classification), and PubMed (https://linqs.org/datasets/#pubmed-diabetes). Detailed statistics are shown in tab:dataset.

**Table 1:** Summary statistics of the 8 network datasets used in this study.

Dataset	Nodes	Edges	Communities	Attributes
Karate	34	78	2	–
Dolphins	62	159	2	–
Football	115	613	12	–
PolBooks	105	441	3	–
PolBlogs	1,490	16,715	2	–
Cora	2,708	5,429	7	Yes
CiteSeer	3,312	4,732	6	Yes
PubMed	19,717	44,338	3	Yes

Karate [49]: The Zachary’s karate club network is a network of friendship among 34 members of a karate club. Over a period of time the club split into two factions due to leadership issues and each member joined one of the two factions.
Dolphins [50]: The dolphin social network was constructed based on the observations recording frequent associations between a group of 62 bottlenose dolphins over a period of 7 years from 1994 to 2001. In this network, dolphins represented as nodes have an edge with each other if they are observed together more often than expected by chance. In previous study, it is generally divided into two communities in terms of sex and age of dolphins.
Football [2]: This is a network of American football games between Division IA colleges during regular season Fall 2000. In the network nodes denote the 115 teams that are divided into 12 conferences, and the edges represent 613 games.
PolBooks [51]: US politics-related books network includes 105 nodes that represent books about US politics sold by the online bookseller Amazon.com. Edges represent frequent co-purchasing of books by the same buyers. The political orientation of these books — liberal, neutral, or conservative — are taken as the ground-truth community label in our experiment.
PolBlogs [52]: The PolBlogs dataset is a directed network of hyperlinks between political blogs collected during the 2004 U.S. election. It includes 1,490 nodes and 16,715 directed edges. The political orientation of each blog is either conservative or liberal.
Cora [53]: The Cora dataset consists of 2,708 machine learning papers classified into one of the seven classes — Case Based, Genetic Algorithms, Neural Networks, Probabilistic Methods, Reinforcement Learning, Rule Learning, and Theory. The citation network consists of 5,429 links. Each publication is described by a 1,433 dimensional 0/1-valued word vector indicating the absence/presence of the corresponding word from the dictionary.
CiteSeer [53]: The CiteSeer dataset consists of 3,312 scientific publications classified into one of the six classes — Agents, AI, DB, IR, ML, and HCI. The citation network consists of 4,732 links. Each publication is described by a 3,703 dimensional 0/1-valued word vector indicating the absence/presence of the corresponding word from the dictionary.
PubMed [54]: The PubMed dataset consists of 19,717 scientific publications from PubMed database pertaining to diabetes classified into one of three classes (“Diabetes Mellitus, Experimental”, “Diabetes Mellitus Type 1”, “Diabetes Mellitus Type 2”). The citation network consists of 44,338 links. Each publication is described by a TF-IDF weighted word vector from a dictionary which consists of 500 unique words.

### Evaluation metrics

To evaluate the community detection performance of baselines and our method, we utilize three widely used performance metrics—Accuracy, Normalized Mutual Information (NMI) [55] and Adjusted Rand Index (ARI) [56]. Accuracy to evaluate division performance from different perspectives. They assesses the community quality by measuring the agreement between the community partition predicted by an algorithm and the ground-truth community partition of the network. Let $𝒫=\left\{{𝒫}_{1},{𝒫}_{2},\ldots ,{𝒫}_{A}\right\}$ be the ground-truth community partition with *A* communities, and $𝒞=\left\{{𝒞}_{1},{𝒞}_{2},\ldots ,{𝒞}_{K}\right\}$ be the community partition detected by an algorithm.

ACC assesses cluster quality by measuring the agreement between the community partition predicted by an algorithm and the ground-truth community partition of a network. ACC is the ratio of the number of correctly predicted samples to whole samples, which is defined as given in Eq. (15).

$$\text{ACC}\left(𝒫,𝒞\right)=\frac{\sum_{i=1}^{N}\delta (P_{i},map(C_{i}))}{N},$$ (15)

where *P*_*i*_ is the actual category label of the *i*-th sample, *C*_*i*_ is the predicted category label of the model on the *i*–*th* sample. The *map* function establishes a mapping between predicted community labels and ground-truth community labels such that the highest accuracy is reached given the partition. δ(x,y) denotes an indicator function defined as shown in Eq. (16):

$$\delta (x,y)=\left\{ \begin{array}{cc} 1 & x=y,\\ 0 & x\neq y \end{array}\right.$$ (16)

$$\text{NMI}\left(𝒫,𝒞\right)=\frac{2\sum_{a=1}^{A}\sum_{b=1}^{K}N_{a,b}\log\frac{N_{a,b}N}{N_{a,\cdot }N_{\cdot ,b}}}{-\sum_{a=1}^{A}N_{a,\cdot }\log\frac{N_{a,\cdot }}{N}-\sum_{b=1}^{K}N_{\cdot ,b}\log\frac{N_{\cdot ,b}}{N}},$$ (17)

where $N_{a,b}=\left|{𝒫}_{a}\cap {𝒞}_{b}\right|$ is the number of nodes in common between the ground-truth community ${𝒫}_{a}$ and detected community ${𝒞}_{b}$, $N_{a,\cdot }=\left|{𝒫}_{a}\right|=\sum_{b=1}^{K}N_{a,b}$, $N_{\cdot ,b}=\left|{𝒞}_{b}\right|=\sum_{a=1}^{A}N_{a,b}$, and *N* is the total number of nodes in *G*.

ARI(𝒫,𝒞)=∑a=1A∑b=1K(Na,b2)−[∑a=1A(Na,·2)∑b=1K(N·,b2)]/(N2)12[∑a=1A(Na,·2)+∑b=1K(N·,b2)]−[∑a=1A(Na,·2)∑b=1K(N·,b2)]/(N2). (18)

The range of NMI and ARI is [0,1]. The value is equal to 1 only if the community partition detected by an algorithm is completely identical to the ground-truth community partition, and 0 for a random partition.

### Baselines

We compare our method with 11 baseline methods listed in tab:models. These methods can be classified into three types according to the network information they exploit. The first type only uses graph structure, including GN [2], LP [25], BGLL [13], and DeepWalk [57]. The second type is the K-means clustering algorithm [59], which only uses node features. The third type uses both the graph structure and node features, including TADW [60], VGAE [29], GUCD [42], DGI [61], ARGA [41], MGAE [40], GRV [62], and SP-AGCL [63]. These studies mostly adopt graph neural networks to learn node embeddings, and then apply the K-means algorithm to obtain node clusters.

**Table 2:** Comparison of information types utilized by different community detection models.

Model	Graph topology	Node attributes
GN	Yes	No
LP	Yes	No
BGLL	Yes	No
DeepWalk	Yes	No
K-means	No	Yes
TADW	Yes	Yes
VGAE	Yes	Yes
GUCD	Yes	Yes
DGI	Yes	Yes
ARGA	Yes	Yes
MGAE	Yes	Yes
GRV	Yes	Yes
SP-AGCL	Yes	Yes
RE-GCN	Yes	Yes

GN [2]: The Girvan-Newman (GN) algorithm detects communities by progressively removing edges with the highest edge betweenness which is defined as the number of shortest paths between node pairs that run through the edge. The edges connecting different communities typically have high edge betweenness, thus removing such edges will separate different groups from one another.
LP [25]: The label propagation (LP) algorithm first initializes every node with unique labels, and then updates their labels iteratively based only on the network structure, where each node adopts the community label that most of its neighbors currently carry.
BGLL [13]: It is an iterative method for unfolding hierarchical communities in large networks. In each iteration, a new network is first built by merging small communities in the previous iteration as a single node, and then larger communities are detected by performing modularity maximization on the new network. A graph partition can be obtained at the top level of the hierarchy.
DeepWalk [57]: DeepWalk transforms the graph structure into node sequences by truncated random walks, and learns node embeddings by applying SkipGram [58] on generated node sequences.
K-means [59]: The K-means algorithm performs node clustering based on the node attributes.
TADW [60]: The text-associated DeepWalk (TADW) model incorporates text features of nodes into network representation learning under the framework of matrix factorization, based on the equivalence between DeepWalk and matrix factorization.
VGAE [29]: The variational graph autoencoder (VGAE) is an unsupervised framework for learning node embeddings, where a GCN encoder is exploited to integrate the topological structure and node attributes into latent node embeddings, and a simple inner-product decoder is used to reconstruct the graph adjacency matrix.
GUCD [42]: It is an unsupervised community detection method for attributed networks, which adopts MRFasGCN [24] as an encoder to derive node community membership in the hidden layer and introduces a dual decoder to separately reconstruct the network structure and node attributes from the derived node community membership.
DGI [61]: Deep Graph Infomax (DGI) is an unsupervised method for learning node representations on graph-structured data, which utilizes graph convolutional architectures to encode the local patch centered around each node, and then maximizes the mutual information between local patch representations and the global graph summary via a noise-contrastive loss.
ARGA [41]: The adversarially regularized graph autoencoder (ARGA) is similar to VGAE. The difference is that an adversarial module is incorporated to discriminate whether the latent node representation is generated from the GCN encoder or from the prior distribution. Once the node representations are learned, the K-means algorithm is applied to perform node clustering.
MGAE [40]: The marginalized graph autoencoder (MGAE) learns node representations by introducing some randomness into the node features and then marginalizes the corrupted features in a graph autoencoder framework, allowing the node content to interact with the network structure.
GRV [62]: The graph representation vulnerability (GRV), an information theoretic-based measure used to quantify the robustness of a graph encoder.
SP-AGCL [63]: A similarity-preserving adversarial graph contrastive learning (SP-AGCL) framework that preserves the feature similarity information and achieves adversarial robustness. The node similarity-preserving view helps preserve the node feature similarity by providing selfsupervision signals generated from the raw features of nodes.

### Experimental results and analysis

#### Performance comparison (RQ1).

To answer RQ1, we compare the performance of RE-GCN with other baselines on both attributed and non-attributed networks. In all experiments, we run the algorithm 30 times on each network, and report the average NMI and ARI for each method. The configuration of hyper-parameters $\alpha_{1}$, $\alpha_{2}$ and $\alpha_{3}$ in this paper are consistent with that in the literature [47]. Experiments show that when $\alpha_{1}\in [0.6,0.8]$, $\alpha_{2}\in [0.15,0.35]$ and $\alpha_{3}=1-a_{1}-a_{2}>0$, RE-GCN reaches the optimal value in most cases, so we set $\alpha_{1}=0.6$, $\alpha_{2}=0.35$, $\alpha_{3}=0.05$.

**Table 3:** Comparison of different community detection methods that only make use of the graph topology on 8 real networks.

Network	Metric	LP	BGLL	GN	DeepWalk	RE-GCN
Karate	NMI	72.76	61.76	61.77	62.06	**100.00**
	ARI	75.31	46.19	46.86	60.89	**100.00**
	ACC	88.23	79.41	82.35	83.41	**100.00**
Dolphins	NMI	60.36	57.60	54.75	63.59	**88.71**
	ARI	47.20	33.74	34.30	68.67	**93.45**
	ACC	74.19	59.67	73.54	77.36	**95.16**
PolBooks	NMI	52.74	56.09	56.15	61.87	**66.88**
	ARI	61.41	66.05	68.24	60.45	**74.90**
	ACC	80.95	78.09	76.53	82.51	**87.61**
Football	NMI	85.73	85.26	87.97	84.52	**91.58**
	ARI	71.09	70.41	77.81	55.85	**86.79**
	ACC	60.91	55.87	66.24	63.92	**86.95**
PolBlogs	NMI	64.56	64.20	65.33	72.61	**75.10**
	ARI	74.94	76.83	75.75	81.66	**83.69**
	ACC	71.55	81.16	76.55	83.44	**92.14**
Cora	NMI	47.17	47.65	47.33	31.75	**52.04**
	ARI	8.82	27.19	21.57	23.93	**46.88**
	ACC	57.49	55.21	61.35	48.41	**74.02**
CiteSeer	NMI	41.68	36.83	38.12	9.66	**41.73**
	ARI	2.33	8.60	8.30	10.11	**42.25**
	ACC	45.21	38.72	40.86	33.71	**71.27**
PubMed	NMI	25.70	23.36	25.37	16.71	**31.10**
	ARI	3.49	11.13	12.43	18.09	**29.88**
	ACC	33.83	35.27	36.24	42.85	**65.54**

**Table 4:** Comparison of different community detection methods that make use of node attributes on 3 real networks.

Network	Metric	K-means	TADW	VGAE	GUCD	DGI	ARGA	MGAE	GRV	SP-AGCL	RE-GCN
Cora	NMI	12.45	36.61	32.92	32.33	41.18	44.96	51.13	49.62	41.85	**52.04**
	ARI	5.83	24.03	25.43	26.39	32.74	35.23	44.81	41.27	32.51	**46.88**
	ACC	49.20	57.41	60.95	66.43	59.08	64.05	63.41	73.35	72.56	**74.02**
CiteSeer	NMI	23.39	32.04	26.01	27.43	31.50	35.02	41.03	33.49	31.47	**41.73**
	ARI	20.43	28.63	20.12	28.02	32.61	34.17	41.42	32.78	30.83	**42.25**
	ACC	54.01	55.86	54.45	58.62	57.98	57.35	63.62	70.09	69.41	**71.27**
PubMed	NMI	26.77	22.42	22.93	26.98	27.73	30.59	28.23	27.42	28.73	**31.10**
	ARI	23.99	17.71	21.38	24.95	31.52	29.50	24.84	28.36	27.19	**29.88**
	ACC	39.84	45.31	63.05	58.72	49.93	66.85	43.95	64.82	63.61	**65.54**

In tab:result_o_attribute, RE-GCN is compared with four different baselines that only exploit the graph topology on both attributed and non-attributed networks. We can observe that the performance of RE-GCN is superior to other algorithms on all networks. Note that the proposed RE-GCN can leverage both the graph topology and node attributes with the help of GCN when refining the structure centers and expanding the pseudo-labeled set. Thus, considering the node attributes is beneficial for improving the performance of community detection. Moreover, for non-attributed networks, compared with DeepWalk, GCN is able to effectively encode the local neighborhood information centered around each node to obtain better node representations for community detection.

In tab:result_w_attribute, RE-GCN is compared with seven methods that can leverage the node attributes on attributed networks. Among them, the K-means algorithm is solely based on the node attributes, while the other methods can leverage both the graph topology and node attributes. These methods achieve better performance than the K-means algorithm, indicating that the graph topology is very necessary for community detection. In addition, our RE-GCN achieves better performance than the other methods that also considers both the graph topology and node attributes. Recall that VGAE, GUCD, ARGA, and MGAE are unsupervised methods with an autoencoder structure, where a common loss is to minimize the reconstruction error for the graph topology and/or the node attributes. DGI is also an unsupervised method which attempts to maximize the mutual information between local node representations and the global graph summary. However, our method is specifically designed for community detection following the spirit of local expansion methods. We first locate and refine the structure centers in a network, each of which can serve as the representative for a potential community and thus is assigned a pseudo label; then the pseudo-labeled set is expanded based on preliminary predictions made by GCN; finally the GCN is trained with the expanded pseudo-labeled set to minimize a classification loss, and then used to infer the community labels for the remaining nodes. Although RE-GCN is also an unsupervised method, good pseudo labels and the corresponding classification loss can more directly enhance the community detection performance.

#### Ablation study (RQ2).

Two key steps of RE-GCN are structure center refinement (ReSC) and pseudo-labeled set expansion (PLSet). To answer RQ2, we compare four variants of RE-GCN. (1) Variant 1 (w/o ReSC and PLSet) directly trains a GCN for community detection under the supervision of the initial structure centers identified by algorithm 1, neither refining the structure centers nor constructing an expanded pseudo-labeled set. (2) Variant 2 (w/o ReSC) does not refine the initial structure centers, but construct a larger pseudo-labeled set based on these structure centers, which is used to train the final GCN for community detection. (3) Variant 3 (w/o PLSet) refines the initial structure centers, but does not expand the pseudo-labeled set before training the final GCN for community detection. (4) Variant 4 is the full model with both ReSC and PLSet. The results are shown in tab:result_o_component.

**Table 5:** Comparison of four variants of RE-GCN.

Network	Metric	w/o ReSC & PLSet	w/o ReSC	w/o PLSet	Full model
Karate	NMI	83.65	83.65	83.65	**100.00**
	ARI	88.23	88.23	88.23	**100.00**
	ACC	91.43	91.43	91.43	**100.00**
Dolphins	NMI	81.15	81.15	81.15	**88.71**
	ARI	87.15	87.15	87.15	**93.45**
	ACC	89.42	89.42	89.42	**95.16**
PolBooks	NMI	55.82	56.67	61.33	**66.88**
	ARI	57.24	59.42	70.37	**74.90**
	ACC	81.09	83.27	85.51	**87.16**
Football	NMI	77.21	79.04	91.01	**91.58**
	ARI	53.66	56.99	85.57	**86.79**
	ACC	78.24	80.33	84.92	**86.95**
PolBlogs	NMI	66.98	66.98	66.98	**75.10**
	ARI	75.51	75.51	75.51	**83.69**
	ACC	81.03	81.03	81.03	**92.14**
Cora	NMI	34.15	40.59	41.62	**52.04**
	ARI	20.52	26.84	33.20	**46.88**
	ACC	41.05	48.27	50.14	**74.02**
CiteSeer	NMI	17.67	27.30	29.15	**41.73**
	ARI	11.46	25.24	27.58	**42.25**
	ACC	37.09	39.72	41.28	**71.27**
PubMed	NMI	21.00	21.77	27.26	**31.10**
	ARI	16.04	18.63	25.32	**29.88**
	ACC	37.53	39.78	45.63	**65.54**

We can roughly obtain a rank of the four variants according to their performance: Variant 1 < Variant 2 < Variant 3 < Variant 4. (1) Variant 1 (w/o ReSC & PLSet) performs the worst. Since it neither refines the initial structure centers identified by algorithm 1 nor constructs an expanded pseudo-labeled set, when some of the initial structure centers are not good, training the GCN with a limited set containing inappropriate seeds will yield unsatisfactory community partition. (2) Variant 2 (w/o ReSC) achieves better performance than Variant 1, but still falls far behind the full model. Note that it expands the pseudo-labeled set on the basis of initial structure centers, without refining them in advance. On the one hand, training the final GCN with an expanded set of pseudo-labeled nodes improves its propagation ability when detecting communities. On the other hand, if inappropriate initial seeds are directly used for expanding pseudo-labeled set, it may mislead the model. (3) The performance of Variant 3 is better than that of Variant 1, but is still lower than the full model. It refines the initial structure centers, which provides high quality seeds for local community detection. However, the final GCN does not have enough propagation ability if it is trained with only the refined structure centers. (4) The performance of Variant 2 is lower than that of Variant 3, indicating that refining the structure centers has a larger impact than expanding the pseudo-labeled set.

Based on the above analysis, we conclude that both structure center refinement and pseudo-labeled set expansion are essential for RE-GCN to achieve its best performance. By updating the initial structure centers, the former step obtains a set of high-quality seed nodes, which lay a good foundation for local community detection. By expanding the pseudo-labeled set, the latter prepares a larger amount of supervision information for training GCN, which helps to improve its label propagation ability.

#### Case study for structure center refinement (RQ3).

**Fig 4:**
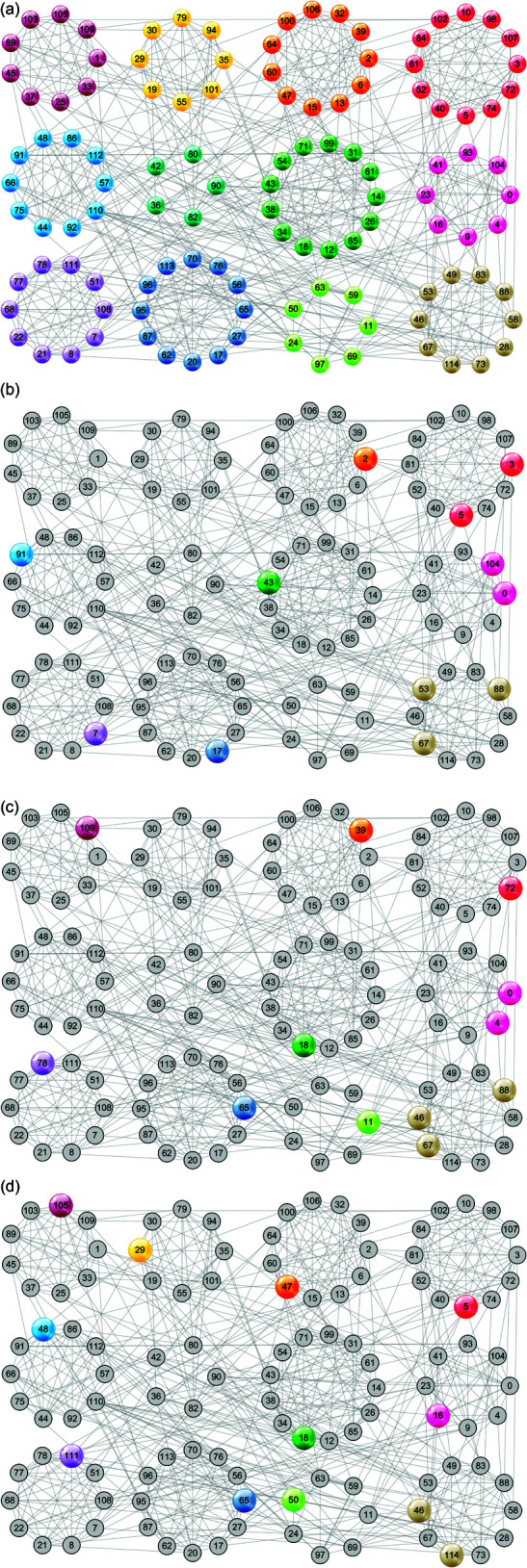
The update process of the structure centers on the Football network when initialized by Algorithm 1. (a) The ground-truth 12 communities in the Football network; (b) Initial structure centers selected by Algorithm 1 are located in 8 communities; (c) 3rd update iteration: structure centers are located in 9 communities; (d) 10th update iteration: structure centers are located in 11 communities.

To answer RQ3, we conduct case studies on the Football network, and visualize the process of structure center refinement. In Fig 4, we first use algorithm 1 to select 12 initial structure centers. As shown in Fig 4(b), some of them are located in the same community, and none of the nodes in 4 communities are selected as structure centers. After 10 iterations of updates, the refined 12 structure centers are scattered in 11 communities, as shown in Fig 4(d).

**Fig 5:**
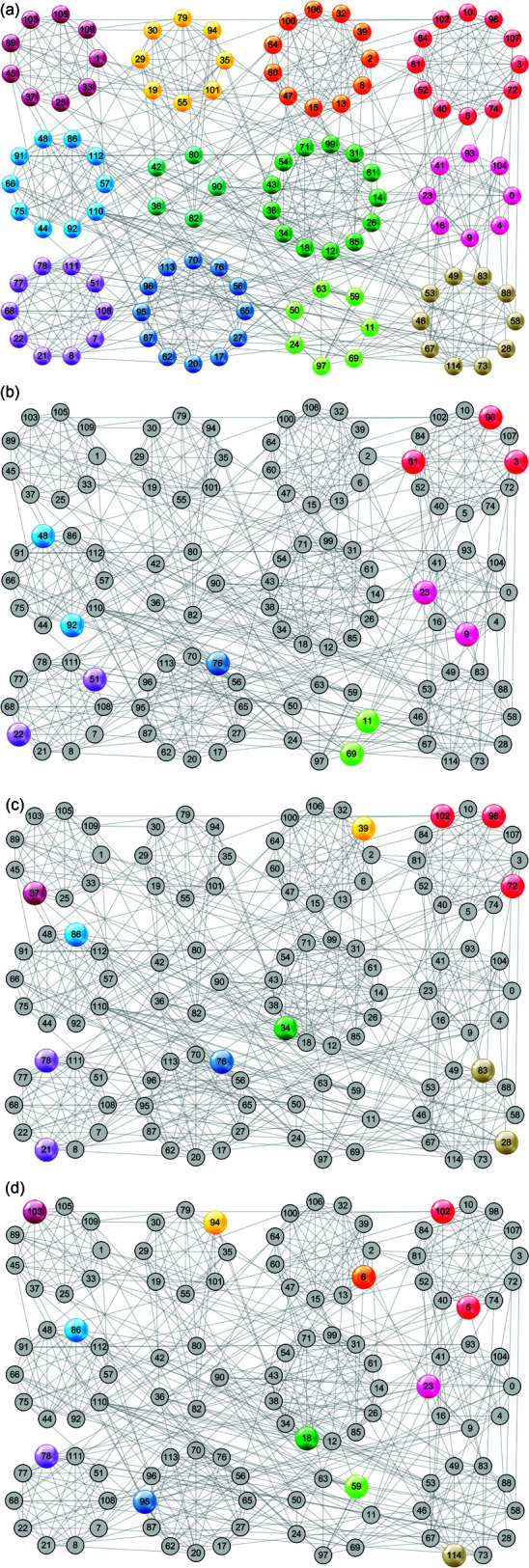
The update process of the structure centers on the Football network when initialized randomly. (a) The ground-truth 12 communities for the Football network; (b) Initial structure centers randomly selected are located in 6 communities; (c) 3rd update iteration: structure centers are located in 8 communities; (d) 11th update iteration: structure centers are located in 11 communities.

In Fig 5 we randomly select 12 initial structure centers, which are located in 6 communities as shown in Fig 5(b). After 11 iterations of updates, the refined 12 structure centers come from 11 communities, as shown in Fig 5(d).

Therefore, no matter the initial structure centers are selected by algorithm 1 or at random, they can be refined to a set of more representative seeds that are scattered in different communities by algorithm 2. That is to say, it can overcome the sensitivity to initial structure centers to some extent, and reduce the adverse effect of inappropriate structure centers on community detection.

**Fig 6:**
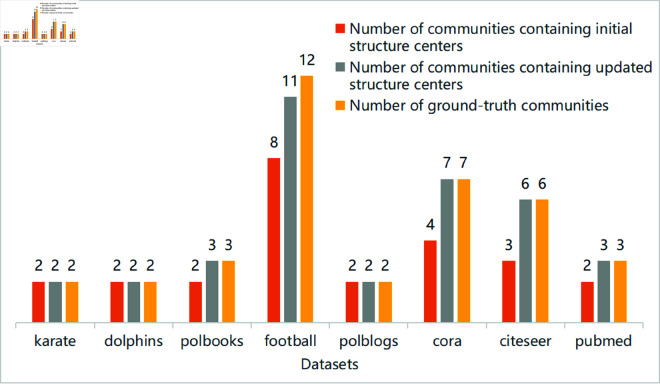
Effect of the structure center refinement process on all datasets.

Fig 6 shows the structure centers refinement analysis for all datasets. The figure shows the variation number of structural centers in each dataset, containing the number of initial structural centers, the number of the updated structural centers, and the number of original communities.

#### Influence of the size of pseudo-labeled training set (RQ4).

**Fig 7:**
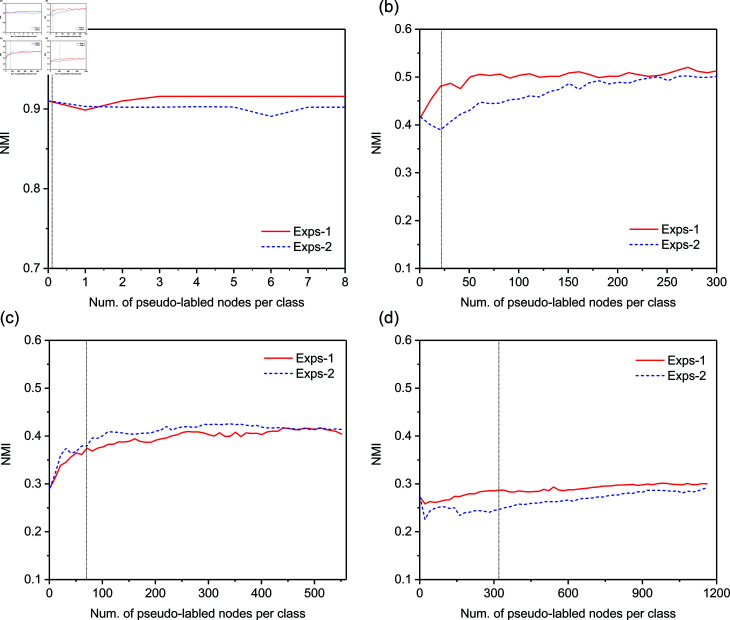
Variation of NMI with respect to the number of expanded nodes per pseudo-label. (a) Football network results; (b) Cora network results; (c) CiteSeer network results; (d) PubMed network results.

**Fig 8:**
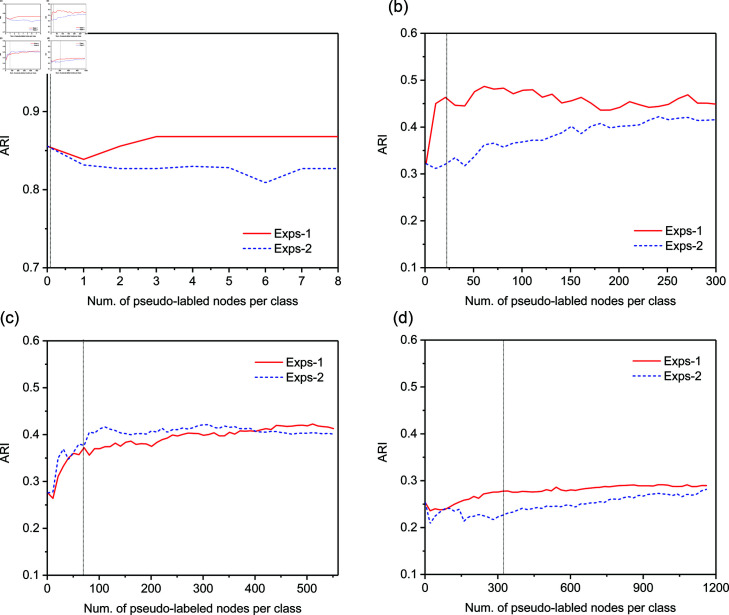
Variation of Adjusted Rand Index (ARI) with respect to the number of expanded nodes per pseudo-label. (a) Football network; (b) Cora network; (c) CiteSeer network; (d) PubMed network.

Finally, we make a comparison of two different expansion strategies specified by Eq 11 and Eq 12 respectively, and investigate how their performance varies with respect to the number of expanded nodes per pseudo label (i.e., τ). Fig 7 and Fig 8 report the variation of NMI and ARI with the increasing of τ respectively, where the vertical line indicates the lower bound of τ.

We can observe that our expansion strategy as defined in Eq 11 outperforms the alternative strategy in Eq 12 on three datasets—Football, Cora, and PubMed, and achieves slightly lower performance on CiteSeer. In all datasets, the community detection performance of RE-GCN with either expansion strategy generally improves as the number of expanded nodes per pseudo-label increases to a moderate size. The reason is that a larger set of pseudo-labeled nodes makes up for the lack of label propagation ability of local graph convolutional filter.

### Computational complexity analysis

The computational complexity of RE-GCN can be decomposed into three main components:

1. **Initial center selection**: This phase computes the structural centrality measures for all nodes. Using Dijkstra’s algorithm with a binary heap for sparse graphs, the time complexity is $O(N^{2}\log N)$. This includes:
  - Computing shortest paths between all node pairs: $O(N^{2}\log N)$
  - Calculating local density ($\rho_{i}$) and relative distance ($\delta_{i}$): $\text{O}({\text{N}}^{2})$
  - Selecting top-*K* centers: O(NlogN)
2. **Iterative refinement**: Each iteration requires:
  - Training a 2-layer GCN: O(|E|) per epoch
  - Generating subgraphs and computing SLP: $O(K\cdot {\bar{n}}^{2})$, where $\bar{n}=N/K$
  - Updating structure centers: $O(K\cdot \bar{n})$
  With *T* iterations (typically *T*<8), the total complexity is O(T·|E|) when ${\bar{n}}^{2}\propto |E|/K$.
3. **Pseudo-label expansion**: This step involves:
  - Calculating affiliation strengths: O(K·N)
  - Sorting nodes for each community: O(K·NlogN)
  For small *K* (e.g., K≤20), this becomes negligible compared to other phases.

The overall complexity is therefore $O(N^{2}\log N+T\cdot |E|+K\cdot N\log N)$. For sparse graphs where |E|∝N, this simplifies to $O(N^{2}\log N)$ when $N\leq 10^{4}$, transitioning to O(|E|) dominance for larger networks.

Memory requirements scale as O(|E|+K·N) due to:

- Storage of graph adjacency and node features: O(|E|+N·d)
- Maintaining community assignments: O(K·N)

where *d* is the feature dimension (typically d≪N).

## Conclusion

In this article, we proposed an unsupervised approach to community detection by structure center refinement and pseudo-labeled set expansion, with GCN as a foundation module which can leverage both network topology and node attributes. It firstly identifies a few structure centers with high local density and large distance from each other based on graph topology. To overcome the sensitivity to initial structure centers, we iteratively refine the structure centers based on both graph topology and node attributes. The refinement process alternates between two steps: obtaining a temporary graph partition by a GCN trained with the current structure centers; updating each structure center to the node with the highest structure importance in the corresponding induced subgraph. To improve the label propagation ability of shallow GCN, we expand the pseudo-labeled set that serves as the supervision information for training GCN. The expansion process selects a few nodes whose affiliation strength to the community is similar to that of its structure center among the subset of nodes that probably belong to the community. The final GCN is trained with the expanded pseudo-labeled set and used to infer the community labels for remaining nodes. Extensive experiments on 8 real networks demonstrate that the proposed approach can achieve better community detection performance than baseline methods on both attributed and non-attributed networks. Additional studies corroborate that both the structure center refinement process and the pseudo-labeled set expansion process contribute to the performance improvement. The refinement process yields a set of more representative structure centers, which can reduce the adverse effect of inappropriate structure centers. And the community detection performance of GCN improves as the number of pseudolabeled nodes increases.

In the future, we would like to explore other techniques for identifying and refining structure centers and expanding pseudo-labeled set when the community definition is different or the community characteristic is vague. In some networks, the community structure may be overlapping, or there may be many weak communities. Under these circumstances, existing strategies may fail to correctly identify all communities.
